# Baseline survey on the scope of education and training for rare bone and mineral diseases

**DOI:** 10.1007/s11657-026-01773-y

**Published:** 2026-09-22

**Authors:** Neveen A. T. Hamdy, Violeta Iotova, Lorena Casareto, Edoardo Gallerani, Alberto Pereira, Luca Sangiorgi

**Affiliations:** 1 Department of Medicine, Division of Endocrinology and Center for Bone Quality, Leiden University Medical Center, Leiden, The Netherlands; 2 Department of Paediatrics, Medical University – Varna, UMHAT “St. Marina”, Varna, Bulgaria; 3 Department of Rare Skeletal Disorders, IRCCS Istituto Ortopedico Rizzoli, Bologna, Italy; 4 Department of Endocrinology and Metabolism, Amsterdam Gastroenterology Endocrinology Metabolism (AGEM), Amsterdam University Medical Center, and University of Amsterdam, Amsterdam, the Netherlands

**Keywords:** Educational and Training programme, Educational tools, Endo-ERN, ERN BOND, Healthcare Professionals, Rare Bone and Mineral Disorders

## Abstract

***Summary*:**

Survey findings from 69 ERN BOND and Endo-ERN health care providers, main stakeholders in advancing the care of patients with rare bone and mineral disorders, reveal the need to develop an accredited, structured, harmonised and accessible cross-border Educational and Training programme in this field.

**Purpose:**

There are disparities in Education and Training (ET) in Rare Bone and Mineral Disorders (RBMDs) among European Member States (MSs). Health care providers (HCPs) from two European Reference Networks play pivotal roles in advancing RBMDs care: ERN BOND for rare bone diseases, and Endo-ERN for rare endocrine diseases. The purpose of this study was to map the ET landscape and identify gaps and unmet needs in this field.

**Methods:**

A 56-questions online survey developed by ERN BOND addressed educational activities, undergraduate and postgraduate ET, competence assessment, and future perspectives in RBMDs. Dissemination was via SurveyMonkey to all ERN BOND (38) and Endo-ERN (78) HCPs.

**Results:**

Collectively, 69 HCPs (60%) representing 20 MSs responded. Most worked in academic institutions, were aged >50 yrs, and had >20 yrs experience. Educational tools most frequently used were PubMed Central searches, phone/email a colleague and attendance of “Meet the expert” sessions and RBMD-related courses. RBMDs were in the undergraduate curriculum of >60% of HCPs. The most popular postgraduate educational tools were formal lectures and learning on the job. Major gaps were specialist ET, and unmet needs were mainly for training workshops/seminars. Nearly all supported the creation of a common educational platform to develop a cross-border educational programme.

**Conclusion:**

Findings from this survey among two ERNs representing the main stakeholders in the excellence of care for patients with RBMDs highlight the need for an accredited, structured, harmonized and accessible cross-border ET programme in this field and inform future initiatives for its development.

## Introduction

Rare diseases (RDs) are seldom encountered in daily practice but collectively represent a significant personal, societal, and economic burden for patients and their families. The main challenges faced by patients with RDs are a lack of awareness, knowledge, expertise, or resources among health care providers (HCPs), resulting in lengthy, costly, and often difficult journeys to diagnosis. ERN BOND (https://ernbond.eu/) is one of 24 European Reference Networks (ERNs) approved and established by the European Commission in 2017 [1], bringing together healthcare professionals with expertise in the care of Rare Bone Disorders (RBDs) representing 38 fully accredited expertise centres from 10 European Member States (MSs), including the United Kingdom until its withdrawal from the EU in January 2020, reaching now 49 HCPs in 19 MSs [2]. The main objective of ERN BOND is to achieve cross-border excellence in health care for patients with RBDs among ERN BOND MSs. This excellence in care can be attained by implementing measures that facilitate multidisciplinary, holistic, and patient-centred approaches in the management of patients suffering from RBDs. For this objective to be achieved, it is paramount to provide excellent and up-to-date education and training to HCP members of this network and to all stakeholders in the management of these disorders, including patients and their families and carers.

A preliminary cursory survey of the breadth, depth, reach and comparability of education and training in the field of RBDs among ERN BOND HCPs suggested disparities in education and training within and among MSs participating in ERN BOND, as well as a distinctive lack of a homogeneous national or European accredited education and training programme in this difficult field of Medicine. It became thus necessary to map the landscape of education and training activities in these RBDs to identify the scope, strengths, weaknesses, gaps, unmet needs, and potential challenges facing the development of such a specific education programme. To this effect, a comprehensive online survey was developed and disseminated among the 38 ERN BOND HCPs, and participation in the survey was extended to the 78 Endo-ERN HCPs. This ERN has a unique structure with eight main thematic groups including one dedicated to the care of patients with rare calcium and phosphate disorders, which significantly affect bone mineralization and thus bone remodeling structure and function [3], and therefore are also cared for by ERN BOND.

The main objectives of the survey were to collect specific information on the scope of existing educational and training activities in the Rare Bone and Mineral Disorders (RBMDs) space among full members of ERN BOND and Endo-ERN, to map and assess the challenges and gaps in education and training in this field and to use the collected data to inform and support the development of a cross-border, accredited, and easily accessible educational programme on RBMDs, in collaboration not only with Endo-ERN but also with other ERNs, and Scientific Societies.

## Methods

The questionnaire was developed in English by the ERN BOND’s Education and Training Working Group and made available online for a 2-month period from September to November 2019 to HCP members of ERN BOND (38) and Endo-ERN (78) through the SurveyMonkey platform. To avoid duplication of data, only one response was accepted per HCP of either ERN.

The complete survey questionnaire (included as Supplementary Material) consisted of 56 questions combining open-ended, multiple-choice, and closed-ended questions. The questions were grouped in 4 sections: background information and educational activities of respondents; scope of education and training in RBMDs, with focus on undergraduate teaching, postgraduate education and training, and educational activities for patients; assessment of education and training competence; and prospective plans for education and training on RBMDs.

Dissemination, data collection and preliminary analysis of the survey were conducted by an external specialized agency “Weber Shandwick” in close consultation with members of ERN BOND’s Education and Training Working Group. Response data from ERN BOND and Endo-ERN were analyzed separately.

## Results

A total of 69 individuals opened and started the survey: 31 of the 38 ERN BOND HCPs (82%), and 38 of the 78 Endo-ERN HCPs (49%). The survey was fully completed by 90% of ERN BOND HCPs and 74% of Endo-ERN respondents who started it.

### Survey section on background information and educational activities of respondents (Questions 1 to 19)

#### Countries represented

**Fig. 1:**
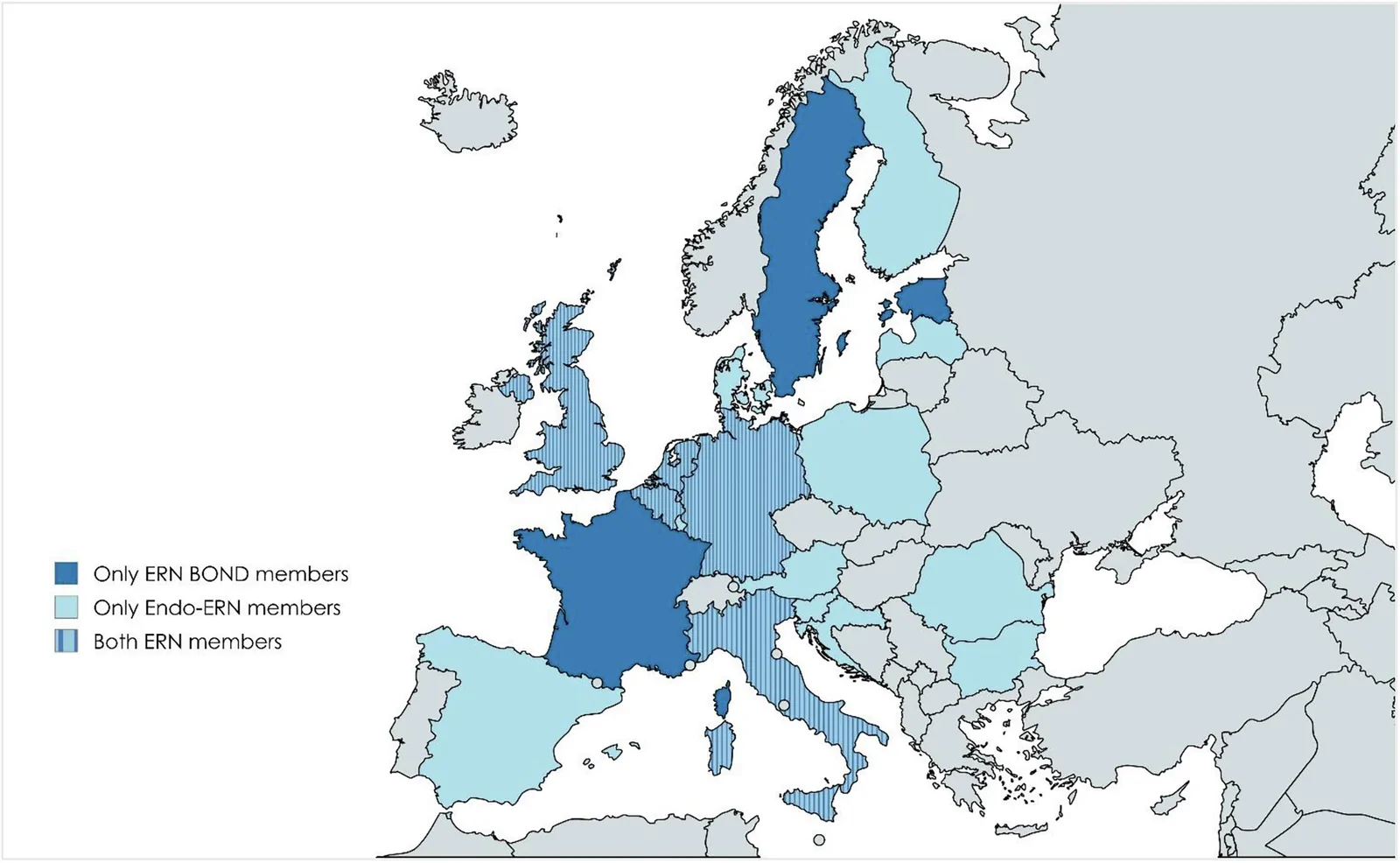
Map of European Member States represented in the survey

The 31 ERN BOND HCPs participating in the survey represented 8 MSs, and the 38 Endo-ERN respondents represented 17 MSs. Collective data thus represented individual non-duplicate entries from 69 full member HCPs from 20 different MSs out of the 28 MSs participating in 2019 (Fig. 1).

#### Type of health care provider institution

More than 2/3 of ERN BOND respondents (68%) and 89% of Endo-ERN respondents worked in an academic hospital setting or in mixed academic/non-academic institutions. None were working in private institutions.

#### Age of respondents

For ERN BOND, the age groups 41–50, 51–60 and >61 years had similar representations (respectively 32%, 35%, and 29%), with most respondents aged >50 years (68%), compared to 50% of Endo-ERN respondents who were overall younger (<50 years, 10% of whom were <40 years).

#### Number of years practicing medicine

As expected by their age ranges, 84% of ERN BOND and 55% of Endo-ERN survey participants had over 20 years of clinical practice, with 37% of Endo-ERN respondents having 11–20 years of experience.

#### Medical specialty of respondents

ERN BOND respondents were primarily pediatric endocrinologists (32%), followed by clinical geneticists (19%) and adult endocrinologists (16%). Other specialties of respondents included orthopedic surgeons (pediatric and adult), rheumatologists, and radiologists. In contrast, all but one Endo-ERN respondents were endocrinologists (58% adult and 39% pediatric).

#### Educational Tools for seeking information on RBMDs

**Fig. 2:**
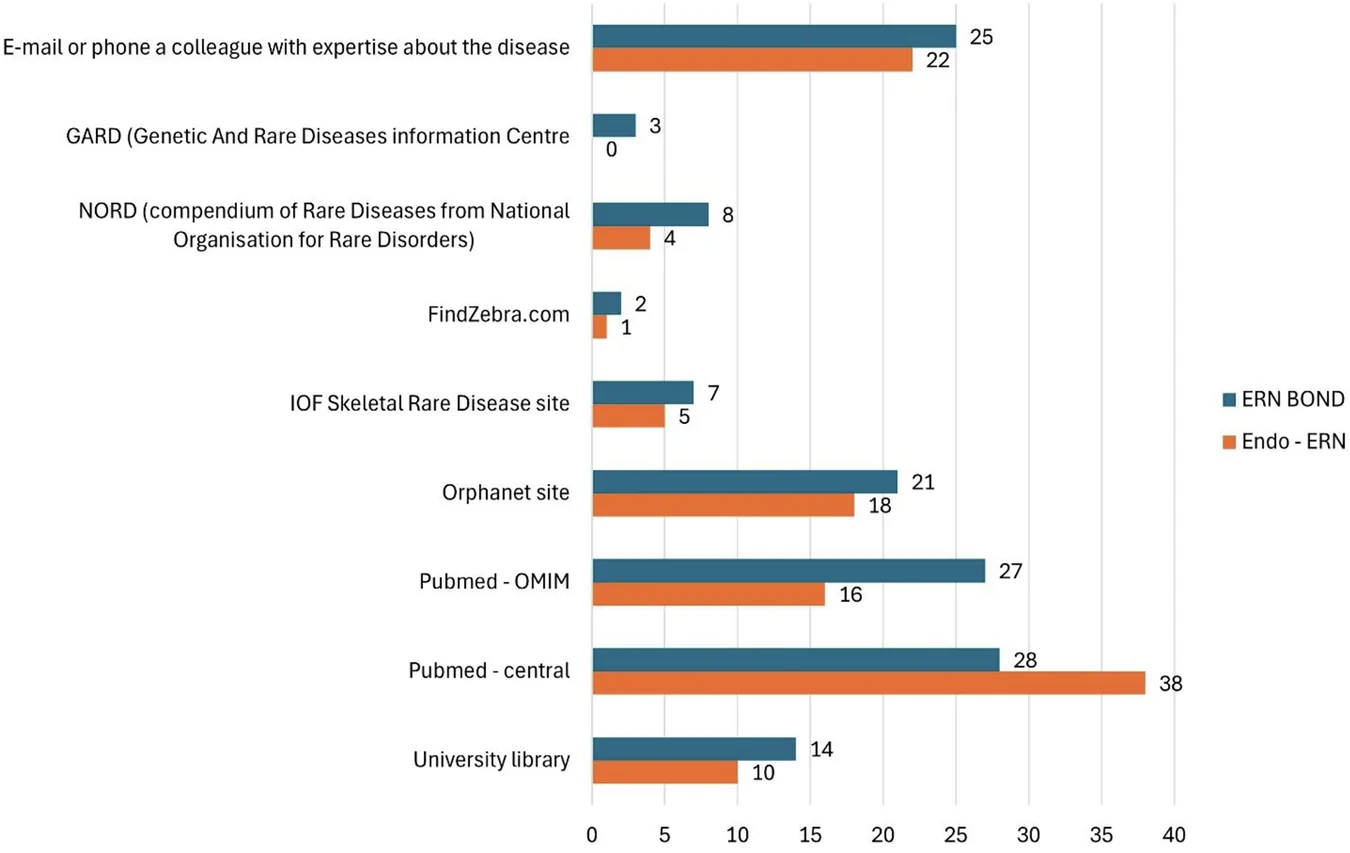
Educational Tools used for seeking information on RBMDs

Both networks relied heavily on PubMed Central for seeking information about RBMDs (90% of ERN BOND and 100% of Endo-ERN). The second most popular tool for both networks was e-mailing or phoning a colleague with specific expertise about the disease (81% ERN BOND and 58% Endo-ERN), highlighting the importance and effectiveness of existing professional networks and short lines of communication within the RBD community regardless of borders. ERN BOND respondents also frequently used disease classification tools such as OMIM and Orphanet (both 68%), as did Endo-ERN respondents to a lesser extent, respectively 47% and 42% (Fig. 2).

#### Membership of scientific societies and attendance of medical congresses

Every ERN BOND respondent reported to be a member of at least one national, European or international scientific society. So were all Endo-ERN respondents but one, most reporting being members of the European Society for Pediatric Endocrinology (ESPE) and the European Society of Endocrinology (ESE). Although some ERN BOND respondents were also members of these societies, they were also members of specifically bone-related scientific societies such as the European Calcified Tissue Society (ECTS), the American Society for Bone and Mineral Research (ASBMR), the International Conference on Children’s Bone Health (ICCBH) and the International Osteoporosis Foundation (IOF). Both network respondents attended international congresses of the mentioned societies, which are at least partly thematically related to RBMDs.

#### Educational activities on RBMDs

**Fig. 3:**
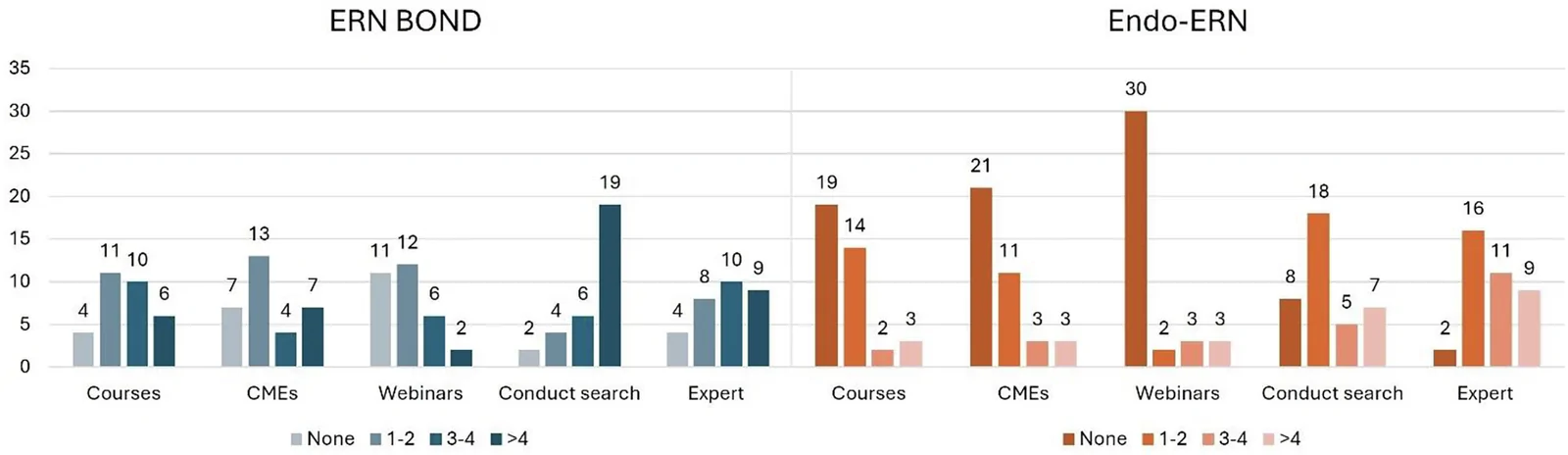
Educational activities on RBMDs in the previous 3 years

For ERN BOND, the most common educational activity conducted for acquiring knowledge on RBMDs was conducting an online search in 94%, with 61% conducting such a search more than 4 times in the previous 3 years. This was closely followed by knowledge being consistently acquired by 87% of respondents by attending “Meet an Expert Session” and by attending courses on RBMDs. The most popular educational activity for Endo-ERN respondents was also attending “Meet an Expert Session” (95%), followed by online searches (79%), and by attending mostly 1–2 courses on RBMDs in the previous 3 years (47%). For Endo ERN, 79% of respondents never attended a webinar over the previous 3 years compared to 35% of ERN BOND respondents; 55% had never completed a CME compared to 23% of BOND respondents; and 50% of respondents had never taken part in a course on RBMDs compared to 13% of BOND respondents (Fig. 3).

#### Involvement of respondents in the organization of meetings, courses and symposia

More than half of ERN BOND respondents (55%) had been generally involved in the organization of meetings, courses or symposia on RBMDs. In contrast, 63% of Endo-ERN respondents had never organized or been generally involved in such activities.

#### Hours per week spent on educational activities

All respondents reported spending some time varying from <2 h to >6 h per week on teaching and learning about RBMDs. Among ERN BOND respondents 84% and 74% reported to be spending on average >2 h per week, respectively on learning and teaching, with 61% spending 2–4 h on learning and 42% on teaching. A similar schedule was reported by Endo-ERN respondents, 76% of whom were also spending on average >2 h/ week on both teaching and learning: 45% 2–4 h/week teaching, and 34% 4–6 h/week learning.

### Survey section on the scope of education and training in RBMDs (Questions 20 to 41)

#### Teaching of RBMDs in Undergraduate Education

Two-thirds of both network respondents reported that RBMDs were included in the undergraduate curriculum of their medical school. Teaching was spread over the 6-year curriculum, varying from <2 h to >6 h/year, with most hours being dedicated to this subject towards the end of the teaching schedule.

For both networks, the most frequently used form of undergraduate teaching of RBMDs was formal teacher-driven lectures in two-thirds of their institutions, with other teaching forms including interactive seminars (<10%), small group tutorials (10% ERN BOND, 14% Endo-ERN), or other forms of teaching such as clinical case-based teaching. Online tools such as e-learning modules, video lectures, webinars or CMEs, were reported to be used by 32% of both networks’ institutions.

#### Postgraduate education and training in RBMDs

According to ERN BOND respondents, education and training in RBMDs was part of postgraduate training mainly in the medical specialties of Endocrinology (68%), Pediatrics (65%) and Genetics (55%), but also in Orthopedics, Rheumatology, Internal Medicine and Rehabilitation. According to Endo-ERN respondents, teaching and training in RBMDs was part of training also mainly in Endocrinology (70%), followed by Pediatrics and Internal Medicine (both 38%), but also in Rheumatology, Clinical Genetics, Orthopedics, and Rehabilitation.

**Fig. 4:**
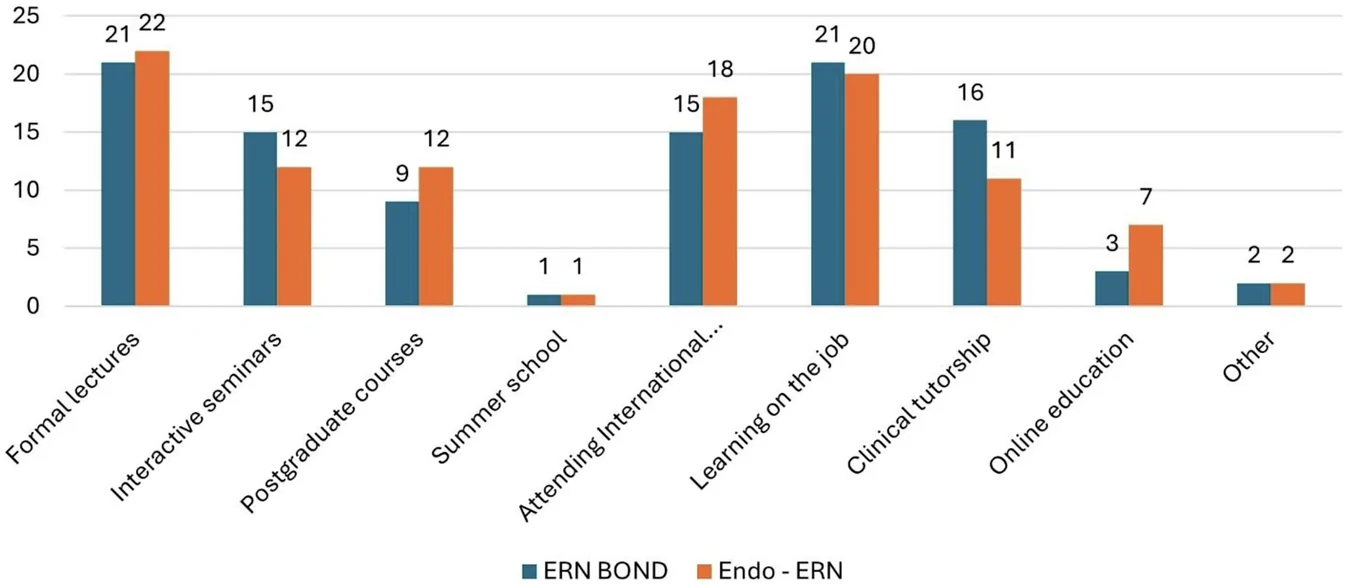
Tools used for postgraduate education and training of HCPs in RBMDs

Most respondents from both networks reported that the most frequent tools used for postgraduate education and training in RBMDs were equally formal lectures (68% ERN BOND, 59% Endo-ERN) and learning on the job (68%, and 54%). These were followed for ERN BOND by clinical tutorships (52%), equally by attending international congresses and interactive seminars (48%), and by attending postgraduate courses (29%). For Endo-ERN, this was equally by interactive seminars and attending postgraduate courses (32%), followed by clinical tutorships (30%) and online education (19%) (Fig. 4).

#### Training measures to address training needs in RBMDs

According to 55% of ERN BOND and 77% of Endo-ERN respondents, training workshops and seminars should be the main training measure to address training needs in RBMDs. For several ERN BOND respondents, a combination of on-campus and online training (28%), and physical exchanges of junior doctors (17%), would also address training needs in RBMDs. On the other hand, none of the BOND respondents thought that webinars or e-learning would address these needs. For Endo-ERN respondents, only 8% considered that webinars or e-learning would be useful, and none recognized physical exchanges of junior doctors, or a combination of on-campus and online training, as relevant to address training needs in RBMDs.

#### Suitable e-learning platforms for RBMDs

A third of ERN BOND respondents (33%) believed that the ESPE e-learning portal was the most suitable e-learning platform for specialists in the field of RBMDs. This opinion is shared by 19% of Endo-ERN respondents, although a majority of whom (58%) believed the combination of the ESPE e-learning portal with the Educational Credential Evaluators (ECE) e-learning platform was the most suitable e-learning platform for specialists in the field of RBMDs. Specifics of a response of “other” from both networks indicated uncertainty or lack of familiarity with existing platforms.

#### Availability and use of Guidelines for RBMDs

ERN BOND respondents reported generally limited guideline availability for use in RBMDs: 1–2 guidelines (38%), 3–4 guidelines (28%), >4 guidelines (17%), with 17% reporting having no available guidelines at all for use in RBMDs. In comparison, 32% of Endo-ERN respondents reported availability of 1–2 guidelines, 6% of 3–4 guidelines and 13% of >4 guidelines, with a further 48% of respondents indicating no guideline availability at all for use in RBMDs.

***Online availability and access to guidelines used by respondents*** was poor for both networks, with less than a quarter of respondents from both networks indicating availability and access to RBMD guidelines through their Centre’s website for local, national or international health care professionals, patients or members of the public.

#### Participation of HCPs in clinical tutorship/mentorship programmes

Most respondents from both networks (79% ERN BOND and 90% Endo-ERN) reported that their Centres were part of national programmes. Respectively, 31% and 32% reported additionally participating in European programmes, and about 10% of each network’s Centres participated in international programmes outside Europe.

#### Role of social media in education in RBMDs

More Endo-ERN than ERN BOND respondents believed that social media play a role in the education of health care professionals (45% vs 29%) or play a role in the education of patients (81% vs 66%).

#### Development of specific educational materials for healthcare professionals

For ERN BOND, 48% of respondents reported the development by their centre of 1–2 specific educational materials for RBMDs for healthcare professionals, compared to only 17% of Endo-ERN respondents. No specific educational material was developed at their respective centres by 69% of Endo-ERN respondents, compared to only 38% of ERN BOND respondents.

#### Collaboration with International Scientific Societies on initiatives regarding educational activities in RBMDs

**Fig. 5:**
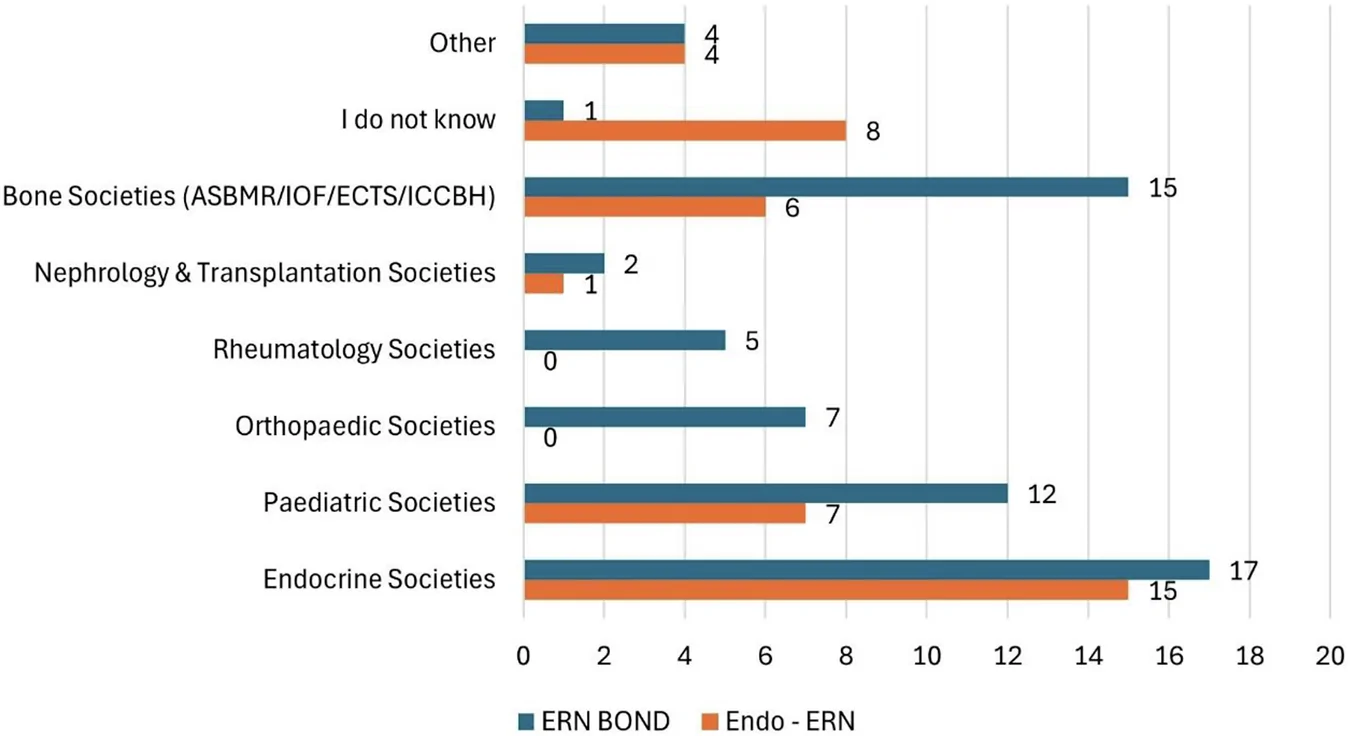
Collaboration with International Scientific Societies on initiatives regarding educational activities in RBMDs

ERN BOND respondents regularly collaborate with International Scientific Societies on initiatives regarding educational activities in the field of RBMDs, with the most frequent collaborations being with endocrine societies (ESE, ESPE) (59%), bone societies (ASBMR, IOF/ECTS/ICCBH) (52%) and pediatric societies (41%). Other ERN BOND collaborations are with orthopedic (24%), rheumatology (17%), and nephrology and transplantation (7%) societies. Endo-ERN respondents also collaborated mainly with endocrine societies (52%), pediatric societies (24%), and bone societies (21%) (Fig. 5).

#### Development of specific educational materials for patients with RBMDs

Specific educational materials for patients with RBMDs were developed by 59% of ERN BOND respondents, mostly 1–2 materials (38%), although 17% reported developing >4 of these educational materials. Most of Endo-ERN respondents (82%) reported that none of these materials were developed, but 11% did report that their centres developed 1–2 specific educational materials and 7% said that more than 4 of these materials had been developed.

Several methods were used by both networks to make disease-specific educational materials available for their patients with RBMDs. The most popular methods were informing patients about patients’ associations and support group websites (ERN BOND 72%, Endo-ERN 54%); handing out locally developed material in the patient’s native language at an out-patient clinic visit (ERN BOND 69%, Endo-ERN 68%); and encouraging patients to contact and join patients associations and support groups (ERN BOND 62%, Endo-ERN 43%). Other methods used included informing patients about the availability of educational material in English on worldwide websites and print as required (ERN BOND 41%, Endo-ERN 36%), or their availability in their native language on their institution’s website and print as required (ERN BOND 28%, Endo-ERN 39%).

#### Involvement in educational activities for patients with RBMDs

The most frequent educational activities for patients ERN BOND respondents were involved in over the previous 3 years were the organization of patient days (79%), the active participation in meetings of national patients’ associations (76%), and extending invitations to patients for lectures, symposia, meetings, and specialized courses on RBMDs (55%). Other activities included the involvement of patient groups in the design of future studies and the writing of grant applications (41%), the placing of educational materials on the websites of patient associations and support groups (38%), and their involvement in the development of clinical protocols (34%). For Endo-ERN respondents, educational activities for patients consisted mainly of involvement in the organization of patients’ days (38%) and active participation in national patient associations meetings (29%).

### Survey section on assessment of education and training competence (Questions 42 to 46)

#### Requirements and frequency of competence assessments for healthcare professionals

Competence assessments were reported to be compulsory by 55% ERN BOND respondents and 43% Endo-ERN respondents. The assessment was reported to be performed yearly (41% ERN BOND, 18% Endo-ERN), or every 5 years (14% ERN BOND, 21% Endo-ERN).

#### Components of competence in RBDM assessment for health care professionals

Among ERN BOND respondents, 29% reported that competence assessment in RBMDs specifically included the number of patients with a diagnosis of RBMD seen by the healthcare professional during the period of training; the number of CMEs collected during attendance of educational activities on RBMDs (17%); the number of specialized procedures performed during the period of training in RBMDs such as bone biopsy or micro-indentation (10%); and results of formal tests and examinations (7%). Other aspects of the competence assessment mentioned included general attendance of educational activities and the assessment of all areas of clinical practice within the appraisal process. Among Endo-ERN respondents, only 5 (17%) reported that competence assessment included the number of CMEs collected during the period of training; 4 (14%) reported that it included the number of patients with RBMDs seen during the training period.

**Fig. 6:**
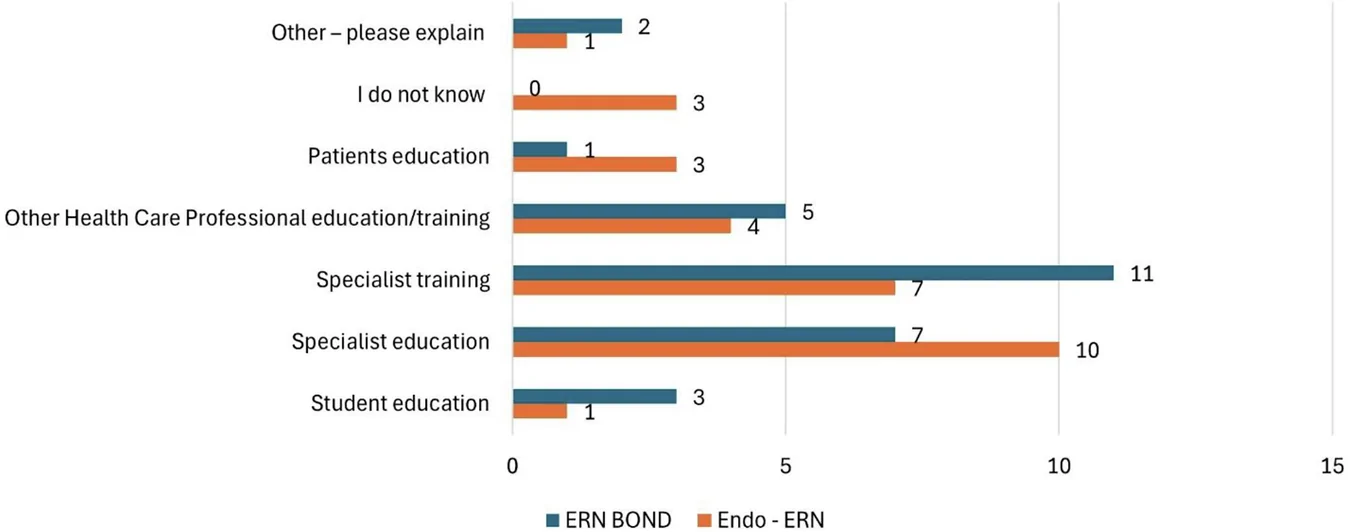
Major gaps in knowledge and competence in RBMDs

***Major gaps in knowledge, competence, and entrusted professional activities in the field of RBMDs*** reported by ERN BOND were specialist training (38%) and specialist education (24%). This was also the case for Endo-ERN respondents, although more cited specialist education (34%) than specialist training (24%) to represent a major gap in knowledge and competence in RBMDs. Other major gaps reported by both networks included gaps in the education and training of other health care professionals involved in the management of patients with RBMDs (ERN BOND 17%, Endo-ERN 14%), and in student education (3 ERN BOND and 1 Endo-ERN respondents) (Fig. 6).

### Survey section on prospective plans for education and training (Questions 47 to 56)

#### Prospective educational plans for health care professionals and patients groups

Among ERN BOND responders, 36% reported that their Centre has a specific educational/training plan for health care professionals as well as for patient associations, support and advocacy groups over the next two years. This number was lower for Endo-ERN respondents, with only 25% reporting that their centre has an education/training plan for health care professionals over the next two years, and 21% reporting having such a plan for patient associations, support and advocacy groups over the next two years. All but one ERN BOND and 2 Endo-ERN respondents were in favor of a common educational platform within ERN BOND and between ERN-BOND and Endo-ERN regarding Rare Bone and Mineral Disorders.

#### Availability of infrastructure/educational platforms to develop an Educational Programme for RBMDs

Whereas 46% of ERN BOND and 43% of Endo-ERN respondents confirmed the availability of infrastructure and educational platforms that would allow the development of a full educational programme in RBMDs, 46% of ERN BOND and 54% of Endo-ERN respondents were not aware whether their centre had the infrastructure or educational platforms to do so or not.

#### Potential number of candidates for an educational programme in RBMDs

Among ERN BOND and Endo-ERN respondents, respectively, 43% and 54% believed the potential number of candidates for a specialized Education and Training Programme in Rare Bone and Mineral Diseases in their countries to be up to 20 candidates, including all specialties and stakeholders involved in the care of patients with these disorders. A minority from both networks quoted higher numbers of potential candidates: 21–30 (*n* = 8), 31–40 (*n* = 7), 41–50 (*n* = 1) and even >50 candidates (*n* = 4).

#### Funding sources for Education and Training initiatives in RBMDs

When asked about sources of funding for Education and Training in RBMDs, networks’ respondents thought that the body that should represent the main funding source for education and training in RBMDs should be the European Commission through ERN BOND (57% ERN BOND, 46% Endo-ERN), or the European Commission through special calls for educational initiatives (25% ERN BOND and 21% Endo-ERN).

## Discussion

The cornerstone of ERNs to meet the objective of cross-border excellence in care of RDs is knowledge generation and dissemination, and the use of the great collective pool of knowledge and expertise to develop clinical pathways and guidelines to facilitate and homogenize the care of patients with RDs across MSs and beyond. An important task of ERNs is also to protect existing knowledge and expertise about RDs, as experts are retiring and there is an urgent need to educate and train younger generations.

The comprehensive online survey, which we report in this paper, was conducted among HCPs from fully accredited ERN BOND and Endo-ERN centres, experts in the care of patients with RBMDs. There was a very good response to the survey, securing good representation from 69 HCPs from the two main ERNs representing complementary stakeholders in the care of patients with RBMDs from 20 of the 28 European MSs at the time of the conduct of the survey. The lower response of Endo-ERN HCPs was probably due to the smaller representation of HCPs in the main thematic group on rare calcium and phosphate disorders, but also possibly due to the lack of awareness, interest, or exposure of HCPs from other Endo-ERNs’ main thematic groups to RBMDs.

Scientific literature has addressed health systems sustainability in the framework of RD educational actions [4], challenges and opportunities of ERNs [5], information needs regarding diagnosis of RDs [6], methods used for education and training in the study of RDs [7], and the use of website systems to support postgraduate medical education [8].

Data from our survey show that there was no significant difference in the perceived major gaps in education and training between ERN BOND and Endo-ERN, although the data did highlight some differences in the methods used for acquiring and sharing knowledge between the two networks, possibly related to the relatively older age and greater years of experience of ERN BOND respondents compared to Endo-ERN respondents. Findings from our survey were also similar to those identified by a survey addressing education and knowledge gaps among Endo-ERN HCPs conducted in 2017 [9]. Our findings were also in line with those of published literature on unmet educational needs in the general RDs space [5, 6].

There are some limitations to our survey. The questionnaire, originally designed for ERN BOND and later extended to Endo-ERN, may not have fully captured the specific context of all respondents. This may have generated a bias due to the possible imbalance in answers provided by Endo-ERN’s respondents because of the smaller gathering of experts specifically dedicated to the care of patients with rare calcium and phosphate disorders in their main thematic group [3].

Another limitation of the survey is that the data reflect the education and training scene in late 2019, although this is also a strength, as the scoping exercise has provided us with a sound basis to work on, and a road map for subsequent developments in education and training activities over the past 5 years that are not captured in this analysis.

Findings from the survey thus highlighted several key issues in education and training on RBMDs that informed and were stepping stones for actions needed to be taken to increase knowledge acquisition and consolidate clinical skills in the care of patients with RBMDs. Positive developments which have already taken place over the past five years were in the form of two main actions. The first action was an ERN BOND knowledge generation webinar programme [8] dedicated to increase knowledge acquisition on RBMDs, which started in May 2021. Since then, this programme has delivered 36 freely accessible webinars, with over 650 participants to the live sessions, and online availability after first release on ERN BOND’s website. A first series of 11 webinars was co-organized by ERN BOND-ECTS in 2023–2024, and 6 webinars were delivered in collaboration with the ERICA project (https://erica-rd.eu/) and EJP RD, the European Joint Programme on Rare Diseases. A second series of webinars is planned for 2026. This followed a similar Endo-ERN knowledge generation programme for webinars for rare endocrine diseases, which successfully generated 30 webinars over the years 2019–2021, although only two of those were dedicated to rare calcium and phosphate disorders [10].

The second action undertaken by ERN BOND was towards consolidation of HCPs clinical skills in the care of patients with RBMDs. This took the form of intensification of the use of ERN BOND’s Clinical Patient Management System (CPMS) Programme. CPMS is the secure, web-based IT platform established by ERNs that allows HCPs to connect and collaborate across national borders on rare and complex cases by facilitating sharing of clinical information during virtual consultations for the diagnosis and management of rare diseases [11]. CPMS represents, in the process, a most valuable and powerful clinical exposure tool that maximizes the otherwise limited possibilities of clinicians’ exposure to rare cases, thereby providing HCPs with invaluable learning opportunities for continuing education and training, thus transforming RD care by bringing experts to the patient rather than patients to the experts.

Survey data also emphasize the need for an educational programme which adopts a uniform structure for developing teaching material, using common formats and platforms and securing funding for development, an aim already pursued by other ERN-related initiatives, such as ERDERA (https://erdera.org/), in the follow-up of the previous EJP RD and ERICA projects. Our data also highlight the need for structured training in the field of RBMDs by means of training exchange fellowships and focused workshops, which has also been increasingly addressed over the past 5 years.

## Conclusion

In conclusion, data from our survey among the two main ERN stakeholders in the excellence of care of patients with RBMDs have captured the scope of existing continuing education and training activities and highlighted the scarcity of clinical guidelines and the major gaps in knowledge and entrusted professional activities in the RBMDs space. The data underline the unfulfilled need to develop Clinical Practice Guidelines for RBMDs and the need for specific competence assessment in the field. The willingness of experts from both networks to support a common educational platform for shared educational projects is very promising, and an important focus for the future will be the development of an Education and Training Programme in RBMDs, which adopts a uniform structure for developing educational material for cross-border use, and of a comprehensive structured clinical training programme in the field also including training exchange fellowships and focused workshops.

## Supplementary information

Hamdy et al Supplementary material
