# Supplementary material for: Baseline survey on the scope of education and training for rare bone and mineral diseases
Source: Arch Osteoporos. 2026 Sep 22;21(1):140. doi: 10.1007/s11657-026-01773-y (PMC13597537; doi:10.1007/s11657-026-01773-y)
Supplement: Supplementary file 1 [file 11657_2026_1773_MOESM1_ESM.pdf]

# ERN-BOND WG8_Survey Education & Training

## ERN-BOND WG8 Survey - Education & Training

**ERN-BOND**

**ERN-BOND is one of the 24 European Reference Networks (ERNs) approved by the European Commission, which gathers expertise in Rare Bone Disorders. ERN-BOND is formed by European health care professionals specialized in the provision of high quality multidisciplinary patient care, scientific research and education and training in the field of rare bone diseases from 38 accredited expertise centres from 10 European Member States (Belgium, Czech Republic, Estonia, France, Germany, Italy, The Netherlands, Portugal, Sweden and The United Kingdom).**

**ERN-BOND's main ambition is to implement measures that facilitate a multidisciplinary, holistic, continuous, patient-centred and participative provision of care for patients suffering from rare bone diseases among ERN-BOND Member States and beyond. It also aspires at supporting patients affected by rare bone diseases and their families in coping with the impact of various aspects of the rare disease on their quality of life. It also aims at increasing the capacity of the patients with a rare bone disease in participating in decision-making regarding their care plan and their life project, in accordance with EUCERD recommendations (2013).**

**Objective of the Survey**

**The objective of this Survey is to collect information on the scope of existing educational platforms and to map and assess the challenges and gaps in Education & Training in Rare Bone & Mineral Diseases within the 10 Member States participating in ERN-BOND, including needs, target groups, contents and existing resources. In consultation with Endo-ERN, which formally covers Rare Mineral disorders, it was decided to extend the scope of the Survey to also include, rare mineral disorders as these intrinsically and structurally also affect the skeleton by leading to rare mineralization disorders and/or altered bone remodelling.**

**The ultimate objective of this exercise is to develop a cross-border accredited, focused, easily accessible, high quality common educational programme to promote innovation of education in the Rare Bone & Mineral Diseases space, in collaboration with Endo-ERN and other ERNs, teaching bodies and Scientific Societies with similar objectives, while identifying the main challenges and the potential solutions to achieve excellent and comprehensive Education & Training in this field.**

**We would be grateful if you could complete this questionnaire before November 1, 2019.**

**If you have any questions about this questionnaire, please contact us at info@ernbond.eu**

## ERN-BOND WG8_Survey Education & Training

### Introduction & Educational Activity Background

**The following questions will provide us with background information and an insight into the educational activities that you have undertaken in the past three years.**

* 1. Please indicate which country you are representing in ERN-BOND/ENDO-ERN: please choose among the  countries in the list.

* 2. Which of the below characteristics classifies you as a health care provider best?

- Academic Hospital - please specify the name below
- Non-academic Hospital - Public/NHS/Peripheral - please specify the name below
- Non-academic Hospital - Private Hospital/Clinic - please specify the name below
- Mixed academic/non-academic - please specify the name below

Please specify the name

* 3. How old are you?

- 21-30
- 31-40
- 41-50
- 51-60
- 61+

* 4. For how many years have you been practicing medicine?

- <10
- 11-20
- >20

* 5. What is your speciality?

- Endocrinology - Paediatric
- Endocrinology - Adult
- Orthopaedics - Paediatric
- Orthopaedics - Adult
- Rheumatology - Paediatric
- Rheumatology - Adult
- Clinical Genetics
- Other Health Care Professional: neurologist, neurosurgeon, ophthalmologist, ENT specialist, maxillofacial surgeon, dentist, rehabilitation specialist, radiologist, physiotherapist, occupational therapist. Please specify

* 6. Which educational tool(s) do you personally use when you seek information about a rare bone & mineral disorder (more than one answer possible)

- [ ] University library
- [ ] Pubmed - Central
- [ ] Pubmed - OMIM
- [ ] Orphanet site
- [ ] IOF Skeletal Rare Disease site
- [ ] FindZebra.com
- [ ] NORD (compendium of Rare Diseases from National Organisation for Rare Disorders)
- [ ] GARD (Genetic And Rare Diseases information Centre
- [ ] E-mail or phone a colleague with expertise about the disease

* 7. Are you a member of one or more National, European or International Scientific Societies?

- ( ) No
- ( ) Yes, please specify.

* 8. Please list Medical Congresses/meetings/symposia you have attended over the past 3 years

* 9. How many courses on Rare Bone & Mineral Diseases did you attend, undertake and/or follow in the past 3 years?

- ( ) None
- ( ) 1-2
- ( ) 3-4
- ( ) >4

* 10. How many CMEs on a Rare Bone & Mineral Disease did you attend, undertake and/or follow in the past 3 years?

- ( ) None
- ( ) 1-2
- ( ) 3-4
- ( ) >4

* 11. How many webinars on a Rare Bone & Mineral Disease did you attend, undertake and/or follow in the past 3 years?

- ( ) None
- ( ) 1-2
- ( ) 3-4
- ( ) >4

* 12. How many times did you conduct a search on a Rare Bone & Mineral Disease did you attend, undertake and/or follow in the past 3 years?

- None
- 1-2
- 3-4
- >4

* 13. How many times did you attend a *Meet an Expert* session during a Congress in the past 3 years?

- None
- 1-2
- 3-4
- >4

* 14. Did you attend, undertake or follow any additional educational activities in the past 3 years?

- Yes
- No
- Yes, please specify which.

* 15. How many meetings/courses/symposia on Rare Bone & Mineral Diseases have you organised or been involved in the organisation of **in your institution** over the past 3 years?

- None
- 1-2
- 3-4
- >4

* 16. How many meetings/courses/symposia on Rare Bone & Mineral Diseases have you **nationally** organised or been involved in the organisation of over the past 3 years?

- None
- 1-2
- 3-4
- >4

* 17. How many meetings/courses/symposia on Rare Bone & Mineral Diseases have you **internationally** organized or been involved in the organization of over the past 3 years?

- None
- 1-2
- 3-4
- >4

* 18. How many hours do you spend on average per week on teaching?

- <2 hours
- 2-4 hours
- 4-6 hours
- >6 hours

* 19. How many hours do you spend on average per week on learning?

- <2 hours
- 2-4 hours
- 4-6 hours
- >6 hours

## ERN-BOND WG8_Survey Education & Training

### Scope of Education & Training in Rare Bone & Mineral Disorders

**The below questions focus on undergraduate teaching.**

* 20. Is the teaching of Rare Bone & Mineral Diseases included in the undergraduate curriculum of your Medical School?

- ◯ Yes
- ◯ No
- ◯ I do not know

21. If yes, how many hours are devoted to teaching Rare Bone & Mineral Diseases over the whole curriculum?

| | None | <2h | 4-6h | >6h |
|---|---|---|---|---|
| First year | ☐ | ☐ | ☐ | ☐ |
| Second year | ☐ | ☐ | ☐ | ☐ |
| Third year | ☐ | ☐ | ☐ | ☐ |
| Fourth year | ☐ | ☐ | ☐ | ☐ |
| Fifth year | ☐ | ☐ | ☐ | ☐ |
| Sixth year | ☐ | ☐ | ☐ | ☐ |

* 22. In which form is undergraduate teaching given?

- ◯ Teacher driven lectures
- ◯ Interactive seminars
- ◯ Small group tutorials
- ◯ Other, please explain.

* 23. Does your Medical School use Online tools (e-learning modules, video lectures, Webinars, CMEs, etc.) in teaching Metabolic/Rare Bone diseases?

- ◯ Yes
- ◯ No
- ◯ I do not know

## Scope of Education & Training in Rare Bone & Mineral Disorders

**The below questions focus on postgraduate education & training.**

* 24. In your Institution, does training in Rare Bone & Mineral Diseases form part of training for the following medical specialties? (More than one answer possible)

- [ ] Internal Medicine
- [ ] Paediatrics
- [ ] Endocrinology
- [ ] Rheumatology
- [ ] Clinical Genetics
- [ ] Orthopaedics
- [ ] Rehabilitation
- [ ] Other, please explain.

* 25. What are the tools used in your Institution for postgraduate education & training in Rare Bone & Mineral Diseases? (More than one answer possible)

- [ ] Formal lectures
- [ ] Interactive seminars
- [ ] Postgraduate courses
- [ ] Summer school
- [ ] Attending International Congresses
- [ ] Learning on the job
- [ ] Clinical tutorship
- [ ] Online education
- [ ] Other, please explain.

* 26. In your opinion, which of the following types of training measures would address training needs in Rare bone & Mineral diseases most efficiently?

- [ ] Training workshops/Seminars
- [ ] Physical exchanges of junior doctors
- [ ] Combination of on-campus/on-line training
- [ ] Webinars
- [ ] E-learning

* 27. In your opinion, which of the following e-learning platforms are suitable for specialists in the field of Rare Bone & Mineral diseases?

- [ ] European Society of Paediatric Endocrinology (ESPE) e-learning portal
- [ ] Educational Credential Evaluators (ECE) e-learning platform
- [ ] Both ESPE and ECE
- [ ] Other (please specify)

* 28. Does your Centre use (local/national/international) guidelines for one or more rare bone & mineral diseases?

- ◯ None available
- ◯ 1-2 (please list)
- ◯ 3-4 (please list)
- ◯ >4 (please list)

Please list the guidelines below

[ ]

* 29. Are guidelines for rare bone diseases used in your Centre available online?

- ◯ Yes (please provide links)
- ◯ No
- ◯ I do not know

Please provide the link

[ ]

* 30. Are your online guidelines for rare bone diseases restricted to your HCP's website?

- ◯ Yes
- ◯ No
- ◯ I do not know

* 31. Are your online guidelines for rare bone diseases available for access nationwide?

- ◯ Yes
- ◯ No
- ◯ I do not know

* 32. Are your online guidelines for rare bone diseases available for access internationally?

- ◯ Yes
- ◯ No
- ◯ I do not know

* 33. Are your online guidelines for rare bone diseases available for access by patients and the public?

- ◯ Yes
- ◯ No
- ◯ I do not know

* 34. Are your online guidelines for rare bone diseases available for access by a other mean?

- No
- I do not know
- Yes, please specify.

* 35. Does your Centre take part in any of the following types of clinical tutorship/mentorship programmes? (More than one answer possible)

- National programmes – please specify
- International programmes within Europe – please specify
- International programmes outside of Europe – please specify

Please specify the above.

* 36. In your opinion, does social media play a role in any of the following?

| | Yes | No | I do not know |
|---|---|---|---|
| Education of health care professionals | | | |
| Education of patients | | | |
| Education of both health care professionals and patients | | | |

* 37. Has your Centre developed specific educational materials for rare bone & mineral diseases for health care professionals?

- None available
- 1-2 (please specify)
- 3-4 (please specify)
- >4 (please specify)
- I do not know

Please specify the above

* 38. Have you or any other member of your HCP collaborated with International Scientific Societies on initiatives regarding educational activities in the field of Rare Bone & Mineral diseases? (More than one answer possible)

- [ ] Endocrine Societies
- [ ] Paediatric Societies
- [ ] Orthopaedic Societies
- [ ] Rheumatology Societies
- [ ] Nephrology & Transplantation Societies
- [ ] Bone Societies (ASBMR/IOF/ECTS/ICCBH)
- [ ] I do not know
- [ ] Other (please specify)

## Scope of Education & Training in Rare Bone & Mineral Disorders

**The below questions focus on the educational activities for patients.**

* 39. Has your Centre developed specific educational materials for patients with Rare Bone & Mineral diseases?

- ○ None available
- ○ 1-2 (please specify)
- ○ 3-4 (please specify)
- ○ >4 (please specify)
- ○ I do not know

Please specify the above.

* 40. How do you make disease-specific educational materials available for your patients? (more than one answer possible)

- ☐ Hand out locally developed material in native language at out-patient visit
- ☐ Inform patients about material available in native language on the institution’s website and print as required
- ☐ Inform patients about Patients Association/Support group websites
- ☐ Inform patients about available material in English on worldwide websites and print as required
- ☐ Encourage patients to contact/join Patients Association/Support groups
- ☐ I do not know
- ☐ Other – please explain

* 41. Have you or any other member of your multidisciplinary team been involved in the following educational activities for patients in the field of rare bone & mineral diseases over the past 3 years? (More than one answer possible)

- ☐ Active participation in annual/biannual meetings of National Patients Associations (formal lectures, discussion groups, Q & A sessions, etc.)
- ☐ Placing of educational materials on website of Patients Associations/Support groups
- ☐ Organisation of Patient Days
- ☐ Extending invitations to lectures, symposia, meetings, courses on rare metabolic / rare bone diseases
- ☐ Involvement of patient groups in design of future studies and writing of grant applications
- ☐ Involvement of patient groups in the development of clinical protocols
- ☐ I do not know
- ☐ Other – please explain

## ERN-BOND WG8_Survey Education & Training

### Assessment of education & training competence among ERN-BOND members

**The below questions are about the assessment of education & training competence among ERN-BOND members**

* 42. Are regular assessments of competence for a health care professional compulsory in your country?

- ○ Yes
- ○ No
- ○ I do not know

* 43. At which intervals are these assessments required?

- ☐ Yearly
- ☐ Every 2 years
- ☐ Every 5 years
- ☐ I do not know
- ☐ Other - please explain

* 44. Are there regular assessments of competence for a health care professional in Rare bone & mineral diseases in your country?

- ○ Yes
- ○ No
- ○ I do not know

* 45. Do these assessments include any of the following?

- ☐ Number of patients with a diagnosis of rare bone disorder seen during the period of training
- ☐ Number of specialised procedures undertaken during the period of training (bone biopsy, microidentation, etc.)
- ☐ Number of CMEs collected during attendance of educational activities pertaining to rare metabolic/rare bone diseases
- ☐ Other - please explain
- ☐ Results of formal tests/examinations
- ☐ I do not know

* 46. In your opinion, where are the major gaps in knowledge/competence/entrusted professional activities in the field of rare bone & mineral diseases?

- ◯ Student education
- ◯ Specialist education
- ◯ Specialist training
- ◯ Other - please explain
- ◯ Other Health Care Professional education/training
- ◯ Patients' education
- ◯ I do not know

## ERN-BOND WG8_Survey Education & Training

### Prospective Plans for Education & Training for participating members of ERN-BOND

**The below questions will focus on the prospective plans for Education & Training for participating members of ERN-BOND**

* 47. Does your Centre have a specific educational/training plan for health care professionals for rare bone diseases over the coming 2 years?

- Yes
- No
- I do not know

* 48. Does your Centre have a specific educational/training plan for Patient Associations/Support groups/Advocacy groups over the coming 2 years?

- Yes
- No
- I do not know

* 49. Are you in favour of a common educational platform within ERN-BOND and between ERN-BOND and Endo-ERN regarding Rare Bone & Mineral Disorders?

- Yes
- No, please state why

* 50. In order to have a common educational platform for Rare Bone & Mineral disorders , would you be willing to share educational material and activities including symposia, day courses, summer schools and other activities such as webinars, webcasts and audio lectures between ERN-BOND and Endo-ERN?

- Yes
- No, please state why

* 51. Would you be willing to participate in the development of an accreditation model for educational/training competence in Rare Bone & Mineral Disorders for network members of ERN-BOND and Endo-ERN?

- Yes
- No, please state why

52. Would you be willing to participate in the development of a ERN-BOND & Endo-ERN full Master Programme in Rare Bone & Mineral Diseases?

- ◯ Yes
- ◯ No, please state why

____________________

53. Does your Centre have the infrastructure/educational platforms to develop such a programme?

- ◯ Yes
- ◯ I do not know
- ◯ No, please state why

____________________

* 54. In your opinion, how many candidates would be potential candidates for specialised education/training in Rare Bone & Mineral Diseases in your country, including all specialties involved in the care of patients with these disorders?

- ☐ Up to 20
- ☐ 21 to 30
- ☐ 31 to 40
- ☐ 41 to 50
- ☐ >50
- ☐ Don't know

* 55. In your opinion, who should be funding initiatives for Education & Training in Rare Bone & Mineral Diseases?

- ☐ Own institution/HCP
- ☐ Medical School to which HCP is affiliated
- ☐ European Commission through BOND-ERN
- ☐ European Commission through special calls for educational initiatives
- ☐ Funding through PAGs
- ☐ Crowd funding initiatives
- ☐ Other – please explain

____________________

56. Are there any other thoughts/suggestions you would like to share on the topic of Education & Training in Rare Bone & Mineral diseases?

____________________
